# Facile green synthesis and characterization of *Terminalia arjuna* bark phenolic–selenium nanogel: a biocompatible and green nano-biomaterial for multifaceted biological applications

**DOI:** 10.3389/fchem.2023.1273360

**Published:** 2023-09-22

**Authors:** Abhijeet Puri, Popat Mohite, Swati Patil, Vijay R. Chidrawar, Yogesh V. Ushir, Rajesh Dodiya, Sudarshan Singh

**Affiliations:** ^1^ St. John Institute of Pharmacy and Research, Palghar, Maharashtra, India; ^2^ Department of Pharmacognosy, Principal K. M. Kundnani College of Pharmacy, Mumbai, Maharashtra, India; ^3^ SVKM’s NMIMS School of Pharmacy and Technology Management, Jadcharia Telangana, India; ^4^ SMBT College of Pharmacy and Institute of Diploma Pharmacy, Nashik, Maharashtra, India; ^5^ School of Pharmacy, Faculty of Pharmacy, Parul University, Waghodia, Gujarat, India; ^6^ Department of Pharmaceutical Sciences, Faculty of Pharmacy, Chiang Mai University, Chiang Mai, Thailand; ^7^ Office of Research Administration, Chiang Mai University, Chiang Mai, Thailand

**Keywords:** antioxidant, antibacterial, anticancer, gel, green synthesis, selenium nanoparticle, *Terminalia arjuna*

## Abstract

Biogenic nanoparticle production is in demand as it is secure, has great promise, and is environmental friendly. This study aimed at green synthesis, characterization, and evaluation of *Terminalia arjuna* selenium nanoparticles (TA-SeNPs) for their antioxidant, antibacterial, anticancer activities, and their incorporation in gel for biomedical applications. The bio-reduction attributes of the *T. arjuna* (TA) bark extract were utilized to fabricate selenium nanoparticles. The TA bark extract is abundant in phenolics (193.63 ± 1.61 mg gallic acid equivalents/g), flavonoids (88.23 ± 0.39 mg quercetin equivalents/g), and tannins (109.46 ± 1.16 mg catechin equivalents/g), which perform as effective capping and stabilizing agents, thus enabling the fabrication of stable SeNPs. The fabrication of TA-SeNPs was corroborated by UV–visible spectra, which exhibited surface plasmon resonance at 291 nm. Transmission electron microscopy (TEM) and scanning electron microscopy (SEM) demonstrated nano-sized spherical TA-SeNPs with an average diameter ranging from 100 to 150 nm. Zeta potential analysis revealed that TA-SeNPs were negatively charged (−26.1 mV). X-ray diffraction presented amorphous TA-SeNPs with a quantification of 82.36 ± 10.2 μg/mL resulting from ICP-AES. The IC_50_ 45.18 ± 0.11 μg/mL for the DPPH assay and 66.51% reducing power capacity values indicated that the TA-SeNPs possessed excellent radical scavenging efficacy. Moreover, the TA-SeNPs exhibited a broad spectrum of antimicrobial activity against potential pathogens. Additionally, the TA-SeNPs exhibited a dose-dependent cytotoxic effect on the MCF-7 breast cancer cell line, with an IC_50_ of 23.41 μg/mL. Furthermore, the TA-SeNP-incorporated gel showed excellent spreadability, extrudability, and consistency with retention of antimicrobial properties and hydrophilic contact angle. As an outcome, TA-SeNPs offer the possibility of the formulation and growth of sustainably designed green SeNPs that can be produced, conserved, and marketed securely across the globe.

## Graphical Abstract

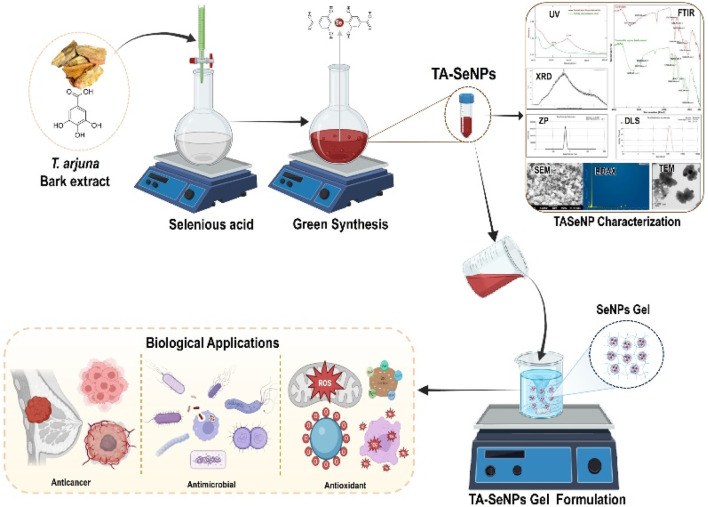

## Introduction

Nanotechnology (NT) has emerged as a desirable technology in the twenty-first century, allowing the production of nanoscale objects with more excellent abilities. The size-dependent properties of nanostructures provide a unique advantage over bulky materials in catalysis, biomedicine, therapeutics, and biosensing (Bayda et al., 2019). Since the tremendous breakthroughs in NT, researchers have been concentrating on nanoparticles (NPs) in therapeutics and medicine. The NT field has shown the ability to transform present diagnostic and treatment techniques (Jayeoye et al., 2021b; Singh et al., 2022b). Green nanotechnology-based metallic NPs aim to develop various scientific products, such as the coating of medical devices (Syukri et al., 2020; Syukri et al., 2021), food preservation, biomedicine, and procedures that are incredibly secure, environmentally friendly, resource-conserving, and waste-free. Phyto-molecules act as stabilizing and reducing agents, making green source-mediated synthesis a potent tool for fine-tuning the size and shape of nanoparticles. It is shown that green biogenic NPs have a substantially higher therapeutic effect than chemically fabricated NPs (Nwabor et al., 2021a).

New selenium (Se) sources, SeNPs, have exceptional *in vivo* bioavailability with reduced selenium toxicity (Bhattacharjee et al., 2019). Derivatives of selenium, such as selenium sulfide, are commonly used in the medical industry to treat microbial infections since selenium (Se) is a powerful antibacterial agent. Since its toxicity is a major concern, scientists have been working hard to improve selenium’s biofunctional qualities while simultaneously decreasing its toxicity. Here, nanotechnology has supplied the most secure technique for reducing the toxicity of selenium and increasing its biofunctionality via green production. The toxicity of Se in its nanoforms is lower, and its antioxidant and antitumor potential is higher than those of its organic and inorganic analogs. The toxicity of selenium oxyanions is reduced when it is present in its elemental form, and *Terminalia arjuna* possesses phenolic compounds, which reduce the Se to its elemental form via the synthesis of TA-SeNPs. The biocompatibility and *in vivo* degradation of SeNPs are significantly better than those of precious metals like silver, gold, and platinum. Compared to organic and inorganic selenium, its nanoforms are less hazardous and have more excellent antioxidant and anticancer potential (Hariharan and Dharmaraj, 2020). In addition, selenium is an essential trace element involved in numerous vital biological processes, and is thus a requirement for all living things. Moreover, fortification of such NPs in topical drug delivery such as with gel, scaffold, and related surgical products is gaining significant attention among researchers due to their excellent antimicrobial properties. Therefore, incorporating reduced metallic NPs in topical formulations is becoming a choice of product due to its compatibility and efficacy, compared to that of antibiotics, which are showing increasing resistance to potential pathogens on a day-to-day basis.

Synthetic processes led to the development of novel solutions due to several drawbacks of chemical and physical NPs. Inorganic SeNPs are less well-absorbed by cells than their organic counterparts, limiting their potential biological applications (Zambonino et al., 2023). Therefore, the biocompatibility and stability of SeNPs can be enhanced by synthesizing them using biomolecules. Green manufacturing of SeNPs has gained significant interest as an alternative method because of several benefits, which include its simple, cost-effective, scalable, and eco-friendly method (Alharbi et al., 2023). Several chemical reduction processes are reported for synthesizing SeNPs (Shoeibi et al., 2017); however, the incorporation of biological processes, including enzymes, microorganisms, and plant extracts, referred to as green chemistry, is imperative for manufacturing SeNPs (Pyrzynska and Sentkowska, 2022). The fabrication of NPs through biological agents, for instance, microorganisms, enzymes, and plant extract, is viewed as a more ecologically friendly alternative to traditional chemical and physical techniques (Pandit et al., 2022). Compared to microorganisms, using plant extracts to fabricate NPs can eliminate the costly steps required to keep cultures alive, compared to conventional chemical and physical processes, using phytoextracts in producing SeNPs, which is the most eco-friendly option (Hano and Abbasi, 2021). This method is typically employed to lessen the hazardous effects and produce environmental friendly chemicals. Fabrication methods have led to novel approaches that address the shortcomings of chemical and physical SeNPs. According to previous reports, various attempts have been made to synthesize NPs using TA extracts. Prasad et al. synthesized SeNPs from TA leaf extract and studied the subsequent impact of SeNPs on arsenite-treated mammalian lymphocytes. Experiments on cell viability (MTT assay) and DNA damage (XTT method) showed that Se-NP-mitigated As (III)-induced cytotoxicity and DNA damage (Prasad and Selvaraj, 2014). Suganthy et al. used TA bark extract to synthesize silver NPs (AuNPs) and studied its antioxidant, anticholinesterase, and anti-amyloidogenic activity for the neuroprotective effect (Suganthy et al., 2018). Although TA bark extract has been used in nanoparticle production, it has not been explored in depth, and the biosynthesis of SeNPs from TA bark extract has not been reported previously.

Nanogels are hydrogel materials that produce intricate three-dimensional networks of cross-linked polymers with a remarkable capacity to hold water because of their nanometer size and ability to withstand dissolution in the surrounding aqueous medium. The choice of polymers used in creating nanogels depends on the desired characteristics, with options including naturally occurring polymers, synthetic polymers, or a mixture of both. The chemical composition of nanogels can be modified to customize their size, charge, porosity, and other attributes for particular uses. At first, nanogels were restricted to spherical particles; however, due to the progress in synthetic approaches, they can now be manufactured in a plethora of shapes. Nanogels have gained recognition as drug delivery devices due to their unparalleled properties, which include exceptional thermodynamic flexibility, high solubilization potential, strong viscosity, and the ability to endure rigorous sterilization techniques. Owing to their hydrophilic nature, nanogels are an excellent option for biomedical applications as they exhibit high biocompatibility and can host many guest molecules (Dalir Abdolahinia et al., 2022). Nanogels have manifested significant potential in multiple domains, including the transmission of chemotherapy drugs and the monitoring and targeting of organs. They have presented fascinating prospects for intelligent drug delivery of nanomaterials, providing targeted therapy and decreased side effects (Soni et al., 2016); Zhang et al., 2018a developed a selenium-containing polyphosphoester nanogel (PSeP) that is responsive to reactive oxygen species (ROS) via a one-step ring-opening polymerization process. Methoxy polyethylene glycol (mPEG) was used as the macroinitiator, and the novel monomer 4-selenoctane-1,8-diyl bis(propylphosphatelane) (Se-COP) was employed. The nanogels were enriched with selenide groups, which provided them with abundant ROS responsiveness upon H_2_O_2_ stimulation. Consequently, the doxorubicin (DOX)-loaded PSeP nanogels manifested inflated behaviors, resulting in the triggered release of doxorubicin. In this method, a swollen release process was also validated by the release mechanisms installed by the Ritger–Peppas power-law model. The results from MTT assays confirmed the non-toxicity of PSeP nanogels and the significant anticancer activity of DOX-loaded PSeP nanogels against A549 cancer cells (Zhang et al., 2018b).

The Combretaceae family, *T. arjuna* Wight and Arn., is widely distributed across Asia and its subcontinents. Several plant bioactive chemicals treat various conditions (Raj et al., 2020). A recent in-depth study showed that tannins, flavonoids, triterpenoids, glycosides, and sterols are found in TA bark extract (Saha et al., 2018). The potential components of TA bark have resulted in its identification as a valuable plant for the green synthesis of SeNPs. However, scant evidence shows the efficacy of NP synthesis using TA bark extract. In this work, we present a simple, eco-friendly approach to fabricate SeNPs from TA bark extract and characterize these NPs via UV, FTIR, XRD, EDX, SEM, TEM, DLS, zeta potential, and ICP-AES and study their biomimetic properties followed with incorporation in hydrogel for quantifying the biomedical applications.

## Materials and methods

Selenious acid (H_2_SeO_3_) was sourced from Sigma-Aldrich Chemicals, India. Gallic acid, 2,2-diphenyl-1-picrylhydrazyl, sodium nitrate, Folin–Ciocalteu’s phenol reagent, ferric chloride, potassium ferricyanide aluminum chloride, trichloroacetic acid, sodium carbonate, Carbopol 940, propylene glycol, and triethanolamine were procured from Loba Chemicals in India. *Escherichia coli* (MTCC 10312), *Klebsiella pneumoniae* (MTCC 3040), *Staphylococcus aureus* (MTCC 1144), and *Bacillus subtilis* (MTCC 1144) were procured from the CSIR—Institute of Microbial Technology’s Microbial Type Culture Collection (MTCC) and Gene Bank in Chandigarh, India. The human breast cancer cell line (MCF-7) and human keratinocyte cells (HaCaT) were acquired from the National Center for Cell Science (NCCS) in Pune, India. All the chemicals used were of analytical grade.

### Plant source, extraction, and phytochemical compositional evaluation

Dr. Jayananda Tosh, Senior Botanist, Research Guide, Sonopant Dandekar Shikshan Mandali (SDSMS), Sonopant Dandekar Arts, V.S. Apte Commerce, and M.H. Mehta Science College, Palghar, verified the plant identity and confirmed its authenticity (VC/154-157/2019-20). The sample specimen is deposited at the Department of Pharmacognosy at St. John Institute of Pharmacy and Research, Palghar, Maharashtra. The TA bark was purged of dust particles by rinsing with deionized water, followed by drying in a hot-air oven (Labline Instruments) set to 40°C. Dried TA bark (20 g) was pulverized and transferred to a beaker containing 500 mL of deionized water. The mixture was heated gently at 40°C until the aqueous solution became brown in a span of 15 min. The mixture was cooled to room temperature and filtered using a Buchner funnel outfitted with Whatman No. 1 filter paper. The acquired extract was centrifuged for 5 min at 1,500 rpm using a centrifuge (Remi C-854/8) and lyophilized and freeze-dried using an SP VirTis Advantage Pro Freeze Dryer (SP Industries, Inc., Warminster, United Kingdom). To detect the bioactive components of the TA bark, a part of the crude TA bark extract was suspended in water and examined through qualitative phytochemical screening.

### Total phenolic, flavonoid, and tannin contents

The quantity of total phenols present in the aqueous extract of the TA bark was assessed with the Folin–Ciocalteu (phenol) reagent, measured using a UV–Vis spectrophotometer (UV/Vis-1800, Shimadzu, Kyoto, Japan) at a wavelength of 725 nm. Outcomes were calculated in terms of milligrams of gallic acid equivalents for each gram of the TA bark extract (mg GAE/g). The aluminum chloride technique was altered slightly from that in past studies to determine the total flavonoid content at 510 nm. The flavonoid content of the extracts was expressed as the equivalent of quercetin (Ismail et al.) per gram of the TA bark extract (mg QE/g). The tannin content of the TA bark extract was determined as reported in the literature (Nwabor et al., 2021b). In brief, 3.0 mL of the extract, 3.0 mL vanillin (4%) in methanol, and 1.5 mL of HCl were mixed and incubated in the dark for 10 min. Subsequently, the tannin content of the samples was measured using a UV–Vis spectrophotometer (UV/Vis-1800, Shimadzu, Kyoto, Japan) at 500 nm and expressed as milligram catechin (CA) per gram of the TA bark extract (mg CA/g).

### Biogenic synthesis of TA-SeNPs

TA-SeNPs were produced with minor changes, as specified in the literature (Dhawan et al., 2021). Briefly, the TA bark extract (10 mL) was drop-wise admixed with 350 mM selenious acid solution (200 ml) under magnetic stirring. To facilitate the induction of the reduction reaction, 2 mL of 400 mM ascorbic acid was added to the mixture at room temperature and stirred for 24 h at 37°C. The reaction mixture was placed on an orbital shaker incubator (CIS-18 Plus, Remi, India) at 200 rpm, incubating in the dark at 35°C for 48 h. The reaction mixture was separated by centrifugation (854/8, Remi, India) for 20 min at 3,500 rpm to remove any remaining unreacted chemicals. The acquired pellet was dried at room temperature in an oven (MSI-5, Meta-Lab, India) for 24 h, followed by washing three times with deionized water and then ethanol. The TA-SeNPs were dissolved in phosphate buffer (pH 7.4), sonicated using an ultra-sonicator (CD4820, Citizen, India), and stored at 5°C for further testing.

### Characterization of TA-SeNPs

The bio-reduction of selenious acid by the aqueous TA bark extract was tracked by observing the color of the reaction mixture. The absorbance of the brick-red TA-SeNPs was measured from 200 to 600 nm using a UV–Vis spectrophotometer (UV/Vis-1800, Shimadzu, Kyoto, Japan). Vibrational and structural characterization of TA-SeNPs was characterized using Fourier-transform infrared spectroscopy (FTIR, Bruker Co., Ettlingen, Germany). The TA bark extract and TA-SeNPs were analyzed at 600–4,000 cm^−1^. The X-ray diffraction (XRD) pattern of the TA-SeNPs was recorded using copper Kα (wavelength of 1.5406 Å) radiation over 0°–60° 2θ at a speed of 2° 2θ/min on a Bruker D8 ADVANCE diffractometer (Bruker AXE, Germany). As reported, the size of the TA-SeNP crystals was determined using the Debye–Scherrer equation (Ontong et al., 2020). The surface morphology and elemental composition of TA-SeNPs were quantified using a scanning electron microscope (FESEM) equipped with an energy-dispersive X-ray spectroscope (JEOL 6390LA/OXFORD XMX N) operating at an accelerating voltage of 0.5–30 kV. The particle size and shape were examined using a transmission electron microscope (TEM; JEM 2100, JEOL, Tokyo, Japan). Furthermore, the size distribution and average diameter of the TA-SeNPs were measured using dynamic light scattering (DLS), while zeta potential was recorded using Zetasizer Nano ZS Ver. 6.34 (Malvern Instruments Inc., Malvern, United Kingdom). The selenium concentrations of TA-SeNPs in parts per million were determined qualitatively using inductively coupled plasma–atomic emission spectroscopy (ICP-AES; SPECTRO ARCOS; Germany).

### Antioxidant activity

The antioxidant activity of TA-SeNPs was evaluated using 2,2-diphenyl-1-picrylhydrazyl (DPPH), and the reducing power was measured by adding a substance to potassium ferricyanide (Fe^3+^), as previously reported (Eze et al., (2022). The radical scavenging activity was calculated using the following equation:

$$Inhibition\;\left(\%\right)=\frac{B}{A}\times 100,$$

where A is the absorbance of the negative control and B is the absorbance of TA-SeNPs.

### Antibacterial activity

The antimicrobial activity of TA-SeNPs was tested using the agar well diffusion method with slight modifications (Singh et al., 2022a). Briefly, *B. subtilis* (MTCC 1144), *S. aureus* (MTCC 1144), *E. coli* (MTCC 10312), and *K. pneumoniae* (MTCC 3040) were cultured in Tryptic soy broth (TSA) supplemented with 40% glycerol and stored at −80 °C. Subsequently, the bacteria were sub-cultured at 37 °C for 18 h–24 h on TSA before being utilized. The bacterial colonies were adjusted to 10^8^ CFU/ML and distributed on TSA plates. Using a cork borer, a 6-mm-diameter well was formed on the TSA plates and then treated subsequently with TA-SeNPs (20 μg/mL and 40 μg/mL), a positive control of ciprofloxacin (40 μg/mL), and a negative control of dimethyl sulfoxide (DMSO). The studies were conducted in triplicate, and the outcomes were reported as the mean and standard deviation.

### *In vitro* biocompatibility assay

The *in vitro* biocompatibility of TA-SeNPs was investigated on fresh red blood cells (RBCs) as reported (Nagime et al., 2023). Briefly, TA-SeNPs, in a concentration range of 100–12.5 μg/mL, were incubated with an equal volume of RBCs. Phosphate buffer and Triton- X were tested as controls separately. After incubation for 1 h at 37°C, treated RBCs were centrifuged at 3,000 rpm for 5 min. The absorbance of the supernatant was measured at 540 nm using a microplate reader (UV/Vis-1800, Shimadzu, Kyoto, Japan). Furthermore, the cytocompatibility of HaCaT cells against TA-SeNPs was investigated using the mitochondrial MTT assay. Concisely, HaCaT cells were grown in a culture medium at a density of 1 × 10^4^ cells/well in 96-well plates as reported (Chidrawar et al., 2023). TA-SeNPs were incubated at a concentration range of 100–12.5 μg/mL for 24 h. After incubation, the spent medium was removed and treated with MTT (0.5 mg/mL) for 2 h, and the absorbance of DMSO-solubilized formazan crystals was measured at 560 nm using a microplate reader (UV/Vis-1800, Shimadzu, Kyoto, Japan).

### *In vitro* anticancer activity

The *in vitro* anticancer effect of the TA bark extract, TA-SeNPs, Se, and doxorubicin was determined using the 3-[4,5-dimethylthiazol-2-yl]-2,5 diphenyl tetrazolium bromide (MTT) assay, as previously reported (Olatunji et al., 2022). Briefly, MCF-7 cells were sub-cultured in Dulbecco’s modified Eagle medium (DMEM) high-glucose medium supplemented with 10% fetal bovine serum (FBS) supplemented with 1% antibiotic–antimycotic solution in a 5% CO_2_ incubator at 37°C. The cells were plated in a 96-well plate at a 1 × 10^4^ cell/well seeding density. A confluence of 80% was applied to the TA bark extract, TA-SeNPs, Se, and doxorubicin; the cells without treatment were tested as untreated controls: MCF-7 cells were inoculated in the culture media only. The plates were incubated in a 37°C, 5% CO_2_ environment for 24 h. A mitochondrial MTT assay was employed to test the cell viability, and the absorption of the formazan crystals was gaged at 570 nm via UV–Vis spectrophotometer (UV/Vis-1800, Shimadzu, Kyoto, Japan). Furthermore, the capability of TA-SeNPs to inhibit cell proliferation was quantified using an indirect *in vitro* scratch assay as reported (Singh et al., 2023). Briefly, the MCF-7 cell monolayer seeded at a density of 5 × 10^4^ cells/well in a 12-well plate was scratched using a 1 mL sterile pipette tip. Subsequently, the cells were washed with PBS (pH 7.4) to remove the cellular debris and replaced with TA-SeNPs (at IC_50_ concentration) in a serum-free medium. The serum-free media was used as a negative control. Images of cell migration were captured at 0 and 36 h using a Carl Zeiss Axio Vert.A1 microscope (Konigsallee, Gottingen, Germany). The residual gap between the migrating cells was measured using the ZEN 2.5 blue edition software of ZEISS.

$$Cell\;migration\;\left(\%\right)=\frac{Distance\;between\;cell\;edges\;at\;0h-After\;36\;h}{Distance\;between\;cell\;edges\;at\;0h}\times 100.$$

### Fabrication and evaluation of the TA-SeNP-incorporated gel

The TA-SeNPs were incorporated with the Carbopol 940 gel base for further biomedical applications. The gel was formulated using 0.5% gelling agent (Carbopol 940) and 0.1% propylene glycol as an emollient and plasticizer. In brief, the required quantity of Carbopol 940 was dissolved in the colloidal suspension of TA-SeNPs along with continuous stirring using a magnetic stirrer followed by the addition of propylene glycol, and the pH was adjusted to obtain gel consistency using triethanolamine. The trapped air bubbles in the gel were removed by storing it overnight in the refrigerator, followed by vacuum. The TA-SeNP-incorporated gel was tested for viscosity, antimicrobial zone of inhibition, spreadability, extrudability, and swelling index, as reported.

### Statistical study

The study was conducted three times, and the sum of the results was the mean ± standard deviation (mean SD). One-way analysis of variances (ANOVA) was used to conduct the statistical analysis, and Dunnett’s multiple comparisons were applied to differentiate between significance levels (*p* < 0.05, *p* < 0.01, and *p* < 0.001).

## Results and discussion

### Phytochemical compositional evaluation

The qualitative phytochemical composition of the TA bark aqueous extract revealed active compounds such as flavonoids, terpenoids, and tannins. Flavonoids and tannins have been reported as capping and reducing agents for the metallic ion, which occurs through their various hydroxy groups and carbonyl moiety (Marslin et al., 2018). Therefore, in this study, selenious acid was reduced and green-synthesized to TA-SeNPs using a phenolic-rich TA bark extract.

### Total phenolic, flavonoid, and tannin contents

Plant polyphenols with hydroxyl and carboxyl functional groups have protonating and absorbing capabilities, which play a vital role in stabilizing metal NPs (Jayeoye et al., 2021a). The results specified total phenolic 193.63 ± 1.61 (mg GAE/g), flavonoid 88.23 ± 0.39 (mg QE/g), and tannin content 109.46 ± 1.16 (mg CA/g) for the TA bark extract, thus suggesting that the aqueous extract of the TA bark contains a substantial quantity of polyphenolics for the green synthesis of TA-SeNPs. The capping behavior of phenolics, tannins, and flavonoids on the surface of metal NPs results in antioxidant action when metal NPs are synthesized using phytochemicals (Kuppusamy et al., 2016).

### Green synthesis of selenium nanoparticles

**FIGURE 1:**
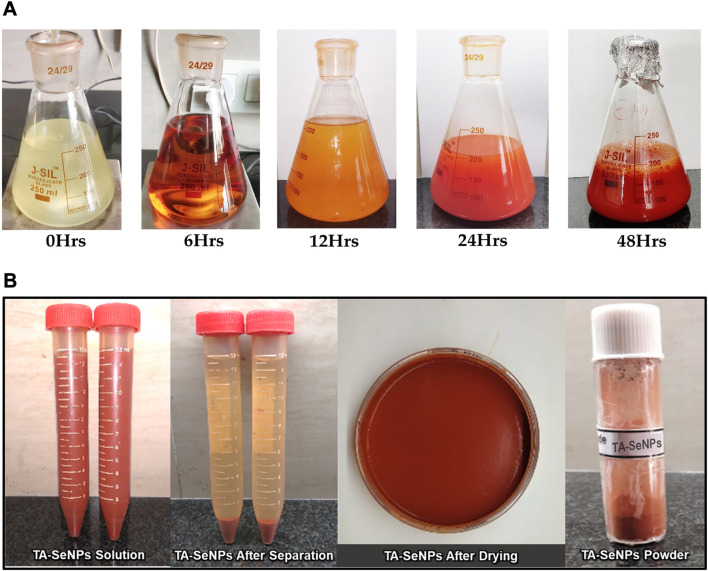
**(A)** Visual examination of *Terminalia arjuna* bark extract–reduced SeNP biogenic synthesis at different time intervals. **(B)** Powder image of TA-SeNPs.

**FIGURE 2:**
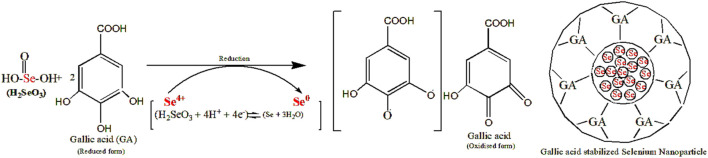
Mechanism for the synthesis of selenium nanoparticles using the *Terminalia arjuna* extract.

TA-SeNPs were fabricated by green synthesis using water as a solvent. The reduction of different concentrations of selenious acid (mM) to TA-SeNPs was accomplished by the phenolic-rich TA bark extract. The reaction was initiated by introducing ascorbic acid, a biocompatible and low-toxicity reducing agent, into the reaction mixture. Initially, the selenious acid solution was colorless, which turned into ruby-red after adding the plant extract. The reaction was initiated by introducing ascorbic acid, a biocompatible and low-toxicity reducing agent, into the reaction mixture. The color change was visually observed as turning reddish-orange (“brick” red color) (Figure 1A). The color change to a “brick” red color is due to the synthesis of SeNPs, which is visually noted after 12 h. The ruby-red solution formed due to the excitation of the surface plasmon resonance and indicated the reduction of selenious acid into elemental selenium. The visible hue shift proved that the selenium ions had been converted into SeNPs. The color of the reaction mixture shifts due to the interaction of the SeNPs with light, which was measured as the surface plasmon resonance (SPR) band by spectrophotometry. The most readily apparent aspect of nanoparticles is their color change as they grow in size. Thus, as the size of the synthesized particle changes, the particle color changes display absorption in the visible area of the spectrum. A powder image of TA-SeNPs for characterization used is shown in Figure 1B. The fabrication conditions of TA-SeNPs were optimized by assessing the pH levels from 7 to 9 and temperatures from 30°C to 50°C. The optimization indicated that the solution transformed from colorless to red or reddish-orange when the temperature was between 35°C and 40°C and the pH level was 8. Previous research has documented that TA bark contains a significant amount of phenolic bioactive gallic acid, catechin, ellagic acid, gallocatechin, and epigallocatechin, which are typical polyphenolics that have the capacity to reduce Se ions. These polyphenolics can bind to the periphery of the selenium ion of selenious acid, hence initiating the fabrication of TA-SeNPs (Alizadeh et al., 2023). It is possible that the hydroxyl groups from polyphenolics might have taken on the role of capping and reducing agents (Khandel et al., 2018). A hypothetical mechanism for the fabrication of SeNPs through a green synthesis process with the help of the TA extract is illustrated in Figure 2.

### UV–Vis spectroscopy analysis of TA-SeNPs

**FIGURE 3:**
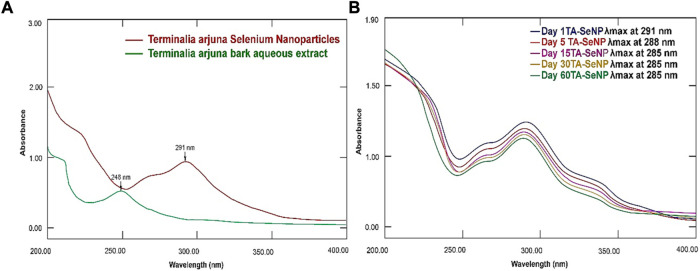
UV–Vis spectrum of **(A)** *Terminalia arjuna* extract, *Terminalia arjuna* bark extract–reduced SeNPs, selenious acid, and ascorbic acid. **(B)** Stability of TA-SeNPs.

A UV–Vis spectroscopy method was employed to analyze the TA-SeNPs and evaluate their kinetic behaviors. The synthesis of TA-SeNPs was validated when the samples were scanned in the 200–800 nm wavelength range. The UV–visible spectra affirmed the fabrication of a ruby-red precipitate of TA-SeNPs after the TA bark extract was added to selenious acid. When the TA bark extract and selenium ions interacted, a color shift occurred, indicating the formation of nano-selenium. As TA-SeNPs increase in size, they demonstrate a gradual change in hue. Variations in particle size result in color changes, which can be quantified by visible-light absorption (Angamuthu et al., 2019). Figure 3A displays the band at absorbance (λ_max_) 255 nm for the TA aqueous bark extract and strong absorbance (λ_max_) at 291 nm for the fabricated TA-SeNPs. A strong surface plasmon resonance (SPR) at 291 nm confirmed the fabrication of TA-SeNPs. A previous study suggests that SeNP maximal absorption was observed between 200 and 300 nm because of their SPR (Fan et al., 2020). The stability of TA-SeNPs was evaluated using UV–Vis spectra (UV/Vis-1800, Shimadzu, Kyoto, Japan). The absorbances were taken at room temperature on days 1, 5, 15, 30, and 60, as depicted in Figure 3B. There were no significant modifications to the peak position for 4 weeks. A minor shift in intensity (288 nm) on day 5 indicated initiation of the aggregation of TA-SeNPs. Later, a shift in intensity to 285 nm on day 30, with a change in shape, was observed, attributed to deformation via nano-surface oxidation. These spectra demonstrated the stability over 2 months of TA-SeNP solutions. The position and form of the SPR band are significantly correlated with particle size, dispersion, and aggregation degree. Specifically, the maximum values of the SPR band shift to longer wavelengths as the particle size increases, and a surge in the peak width correlates with an intensification in particle dispersity, showing excellent stability (Vijayakumar and Ganesan, 2012). Notably, the nanohybrid system keeps its appearance and distinctive absorption band after several weeks of storage in a refrigerated environment.

### FTIR analysis of TA-SeNPs

**FIGURE 4:**
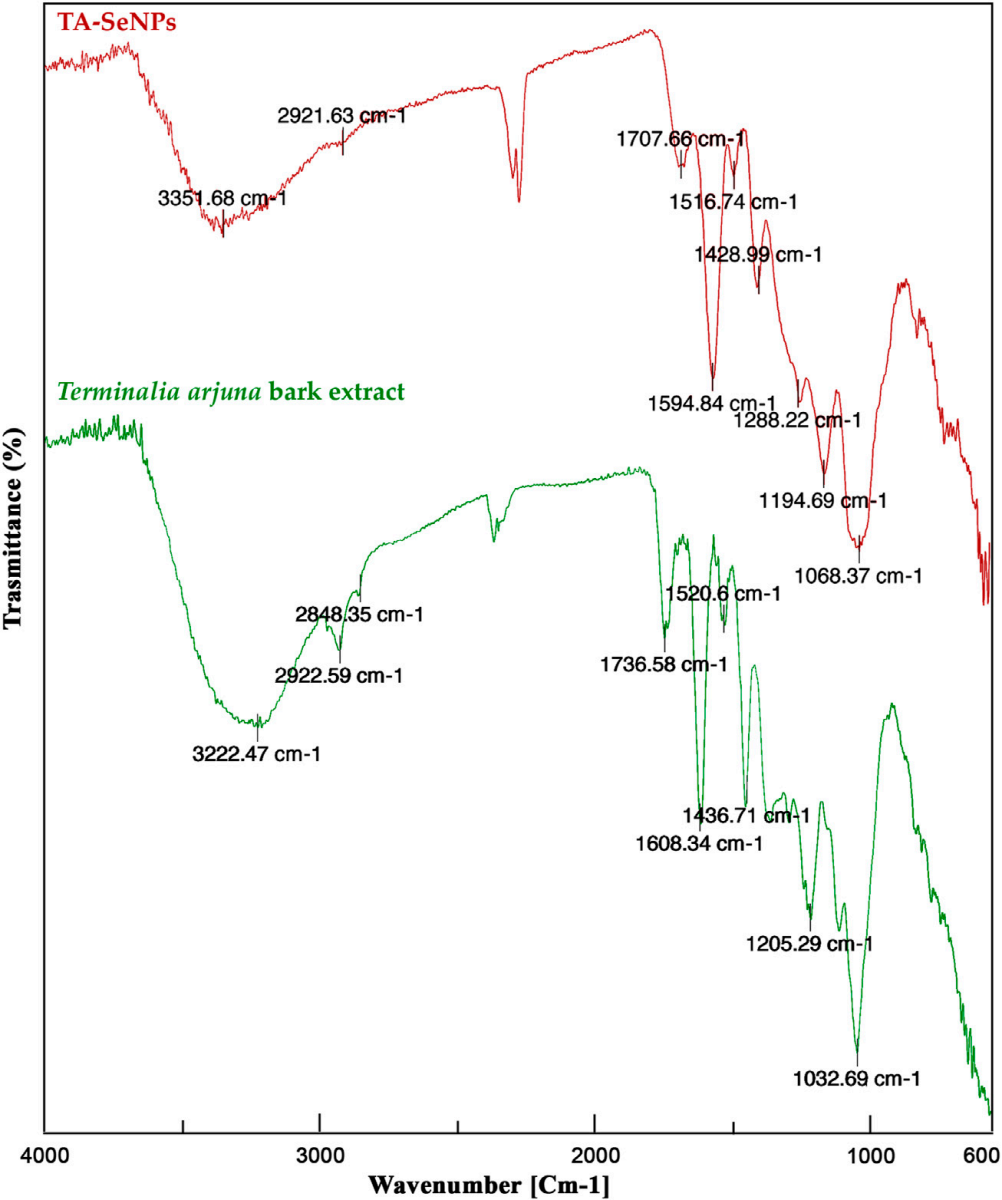
Fourier-transform infrared spectroscopy analysis of the *Terminalia arjuna* bark extract and TA-SeNP.

The process of reduction and fabrication of TA-SeNP by phyto-molecules of the TA bark extract is validated by FTIR analysis, as depicted in Figure 4. Through Fourier-transform infrared spectroscopy, the vibrational frequencies of chemical bonds can be assessed in order to recognize the functional groups on the surface of SeNPs (Gunti et al., 2019). The FTIR spectrum of the TA bark extract depicted multiple intense peaks at ∼3222.47 cm^−1^, ∼2922.59 cm^−1^, ∼2848.35 cm^−1^, ∼1736.58 cm^−1^, ∼1608.34 cm^−1^, ∼1436.71 cm^−1^, ∼1365.28 cm^−1^, ∼1205.29 cm^−1^, ∼1032.69 cm^−1^, and ∼882.23 cm^−1^, which links to the presence of the –OH group, C–H stretching vibration of aliphatic, O–H of carboxylic acid, C=O carbonyl stretch, aromatic C=C, bending in alkyls CH_3_, C–H, and R–O–R (ether), as well as C–H bending of flavonoids in the plane of superposition, stretching vibration of C–C, and phenolic group OH bending, respectively. The broad vibration peak at ∼3351.68 cm^−1^ of O–H stretching in TA-SeNPs indicates alcohol and phenols, where, during biosynthesis of TA-SeNPs, the broad, intense peak at ∼3222.47 cm^−1^ of the TA bark extract was shifted to ∼3351.68 cm^−1^ for TA-SeNPs. Through hydrogen bonding, selenium is thought to have interacted with the hydroxyl group from the TA bark extract, which enabled the biosynthesis of TA-SeNPs. A peak at ∼1736.58 cm^−1^ carbonyl (C=O) stretching vibrational absorption of flavonoid (Wen et al., 2021) of the TA bark extract shifted to ∼1707.66 cm^−1^ in TA-SeNPs, specifying that a carbonyl C=O stretch has enabled the synthesis of TA-SeNPs. The prominent intensity peak at ∼1032.69 cm^−1^ of the TA bark extract was shifted to ∼1068.37 cm^−1^ in TA-SeNPs, which represents the Se–O stretching vibration (Kannan et al., 2014), and the disappearance of the peak at ∼2848.35 cm^−1^ indicates the involvement of alcohols and alkanes in the capping of selenium in synthesizing TA-SeNPs. Thus, FTIR studies indicated that phenolics predominated on the surface of TA-SeNPs. The transition in the peaks seen in TA-SeNPs shows that polyphenols from the TA bark extract have helped produce TA-SeNPs through reduction and stabilization.

### XRD analysis of TA-SeNPs

**FIGURE 5:**
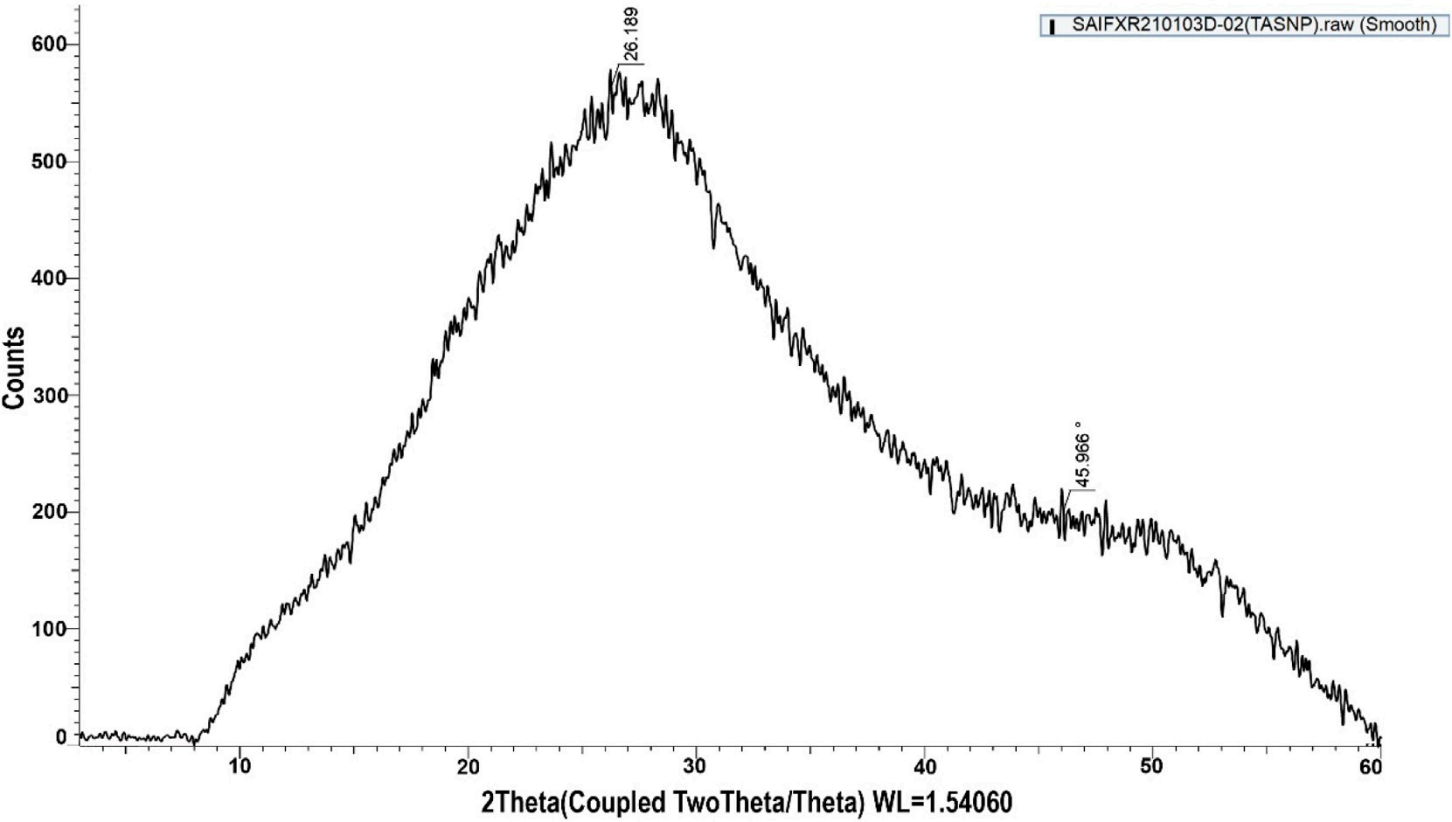
X-ray diffraction spectra of *Terminalia arjuna* bark extract–reduced SeNPs.

The XRD pattern of fabricated TA-SeNPs is depicted in Figure 5, which displays broad peaks between 2θ = 20°–30° at low angles. The X-ray pattern does not feature any distinct Bragg’s peaks, instead displaying a broad peak, as other researchers noted that the amorphous region is predominant in synthetically created SeNPs. The data suggest that fabricated TA-SeNPs are amorphous, consistent with prior findings (Mellinas et al., 2019). Since oxygen is present in the medium reaction, the other peaks in the XRD spectrum may represent the diffraction peaks of partially oxidized selenium oxide (Vu et al., 2022). Amorphous selenium exhibits a red hue (Dwivedi et al., 2011), as confirmed in the TA-SeNP solution. The selenium colloids’ red color indicated their amorphous or monogenic nature since trigonal selenium is black (Zhang et al., 2004; Gunti et al., 2019). However, the XRD pattern confers to the trigonal phase of selenium, which appears as diffraction peaks representing these stated phases of Se, with lattice constants a = 4.366 A° and c = 4.956 A° (JCPDS file no. 06-362) (Alagesan and Venugopal, 2019). The average particle size of TA-SeNPs was calculated to be between 110 and 120 nm.

### Morphology, energy-dispersive X-ray, particle size, and zeta potential analysis of TA-SeNPs

**FIGURE 6:**
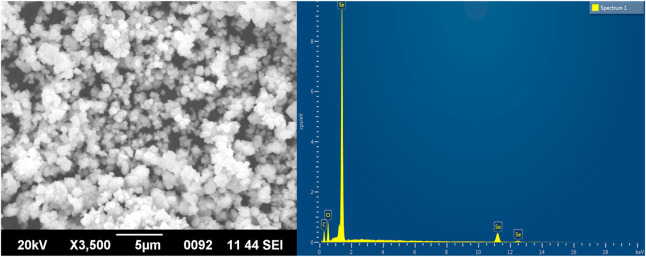
Scanning electron microscopy images and EDX analysis of *Terminalia arjuna* bark extract–reduced SeNPs.

SEM demonstrated that TA-SeNPs were spherical with a smooth surface. Particle size, calculated through the SEM photomicrograph, indicated a particle size range of 50–150 nm distributed by aggregation (Figure 6). The TA bark extract may contain different functional groups that bind to and nucleate selenium ions, which could explain why the SeNPs are found there. Only a small fraction of the best available metal ions form complexes during nucleation, indicating metal ion aggregation. Previous research has shown that agglomerated NPs have a higher biological activity (Kalishwaralal et al., 2016).

Furthermore, the EDX analysis demonstrated a distinctive absorption peak at 1.4 keV, with some weak signals at 11.2 and 12.4 keV, which corresponds to the element Se in TA-SeNPs, with a weight of 59.59% (Figure 6). Additionally, medium signals were presented at 0.4 and 0.5 keV, which showed oxygen (O) and carbon (C), with a weight of 15.56% and 24.84%, respectively, showing the role of phyto-molecules as a reducer was linked to the surface of the TA-SeNPs. As reported by Sharma et al., the high selenium content in the spectrum is evidence of the purity of the synthesized selenium metal (Sharma et al., 2014). There are oxygen and carbon peaks in the spectra of TA-SeNPs, suggesting the existence of alkyl chain stabilizers. The presence of oxygen in the spectrum may result from functional group-containing stabilizers (Ramamurthy et al., 2013).

**FIGURE 7:**
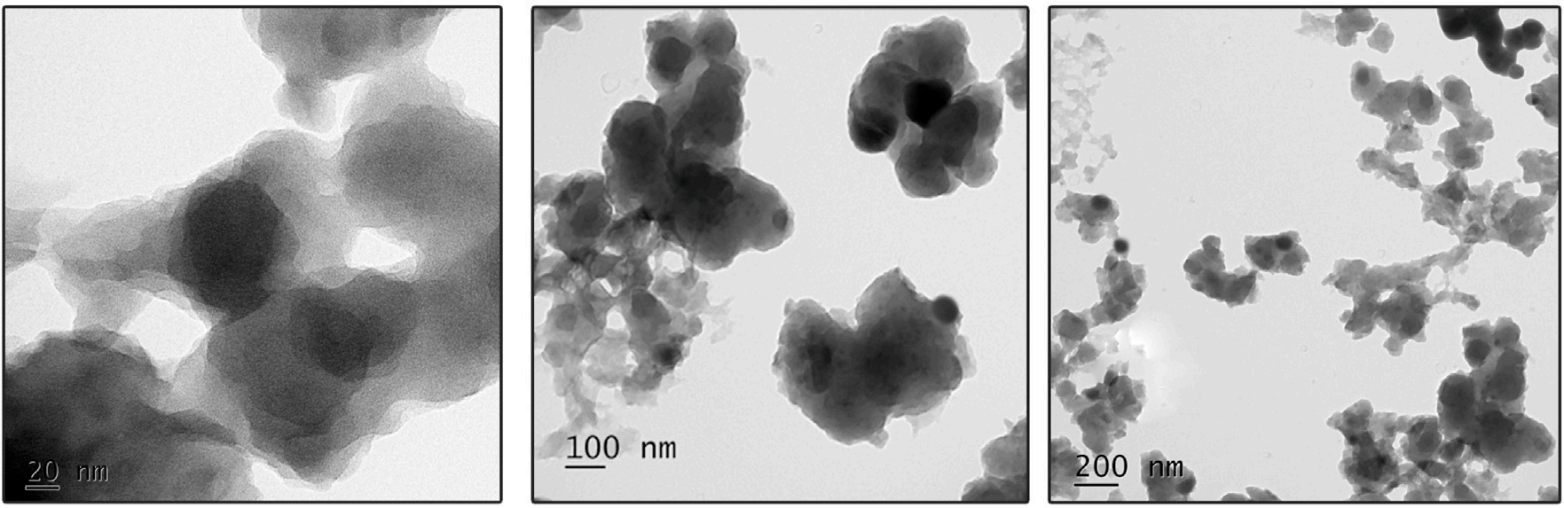
Transmission electron microscopy images of *Terminalia arjuna* bark extract–reduced SeNPs.

Furthermore, the particle size, size distribution, and morphology were recorded using TEM, as depicted in Figure 7. Bio-fabricated TA-SeNPs were mono-scattered, spherical, and nanoscale (20–200 nm) in size distribution, with an average size of 115 nm. Due to several polyphenolics with reduction potential, the sizes and shapes of the produced TA-SeNPs were independent of the TA bark extract used in synthesis (Kora, 2018). As a result, the TA-SeNPs generated in this way may be stabilized and partly aggregated, which may cause sedimentation at a later time. A previous study indicated that metallic particles might get agglomerated due to an excess concentration of reducing extract (Balavijayalakshmi and Ramalakshmi, 2017).

**FIGURE 8:**
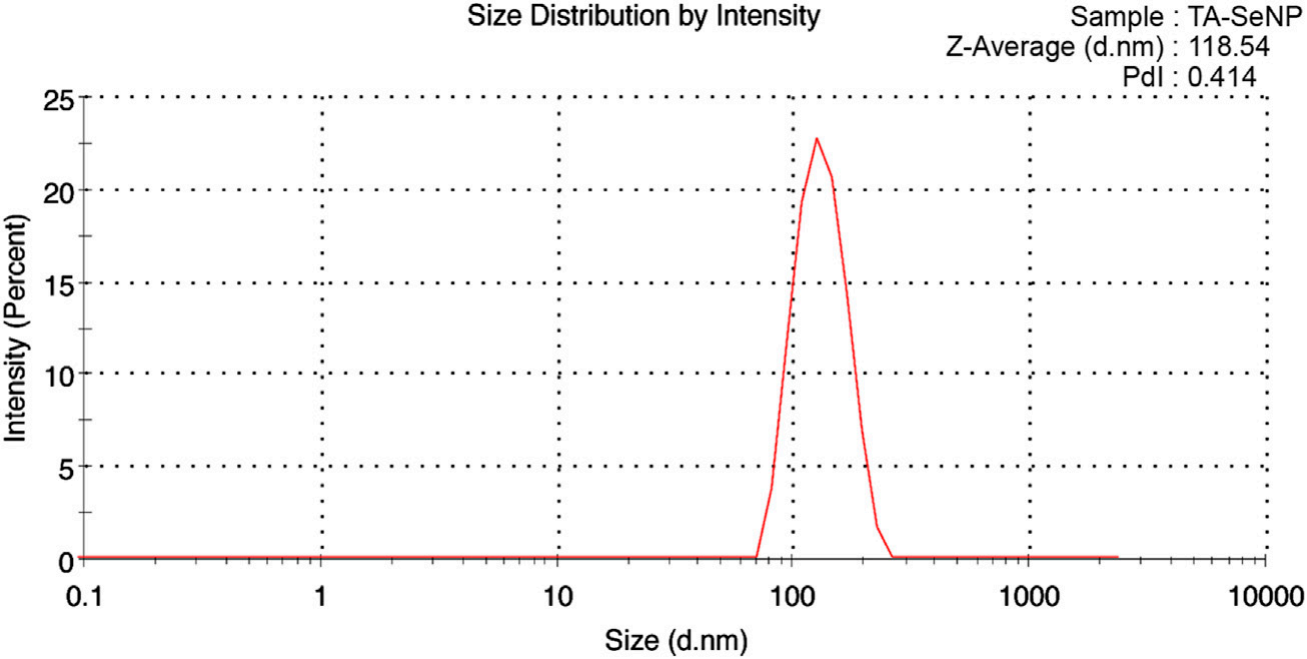
Particle size distribution analysis using dynamic light scattering analysis of *Terminalia arjuna* bark extract–reduced SeNPs.

The particle size was measured by DLS analysis, as depicted in Figure 8. It was found that the average size of TA-SeNPs is 118.54 nm, and their PDI is 0.414, demonstrating their homogeneity and uniform dispersion. As measured by TEM and DLS, the particle size differs; this may be due to the effect of Brownian motion; the size determined by DLS is more significant than that estimated by TEM. According to DLS measurements, the particle diameter is only affected by the solvation shell produced on the selenium core by the TA bark extract (Zhang et al., 2018a).

**FIGURE 9:**
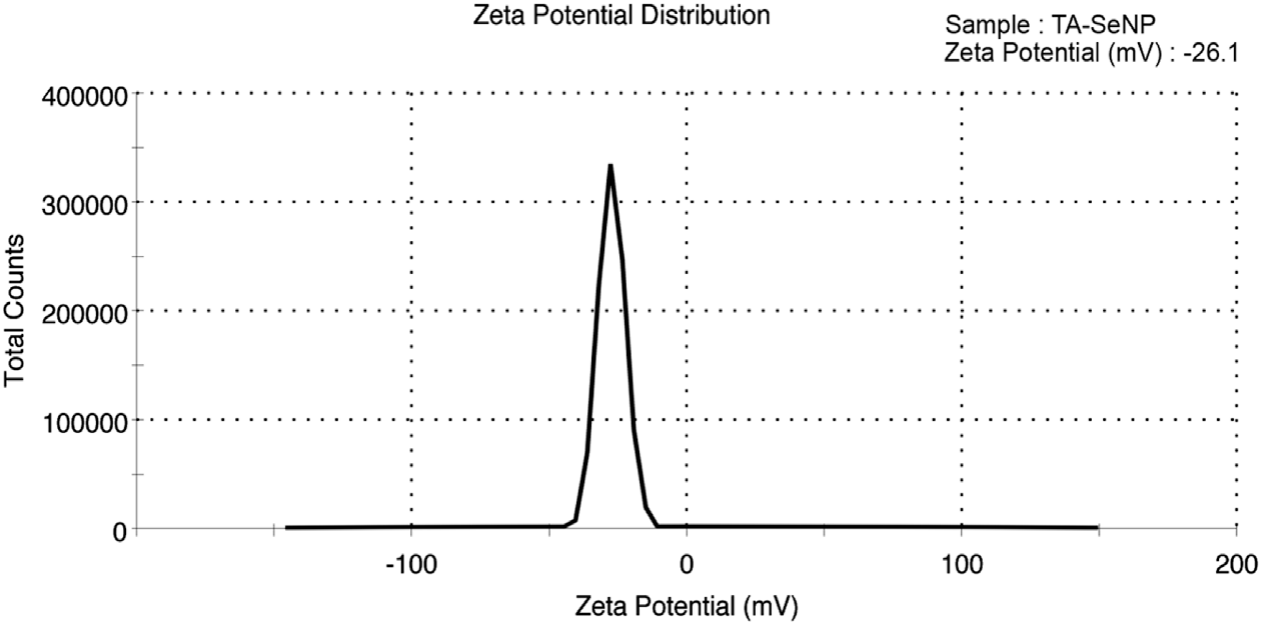
Zeta potential of *Terminalia arjuna* bark extract–reduced SeNPs.

The zeta potential analysis showed that the fabricated TA-SeNPs were negatively charged (−26.1 mV), suggesting a stable fabricated form (Figure 9). The reducing agent oxidized the polyphenolics in the TA bark extract, creating negatively charged particles. The higher negative charge on the SeNP surface signifies the superior stability due to the intrinsic capping by phyto-molecules. This suggests the presence of large electrostatic forces between the green synthesized SeNPs. Previously, if all particles in a suspension had a negative or positive zeta potential, they tended to reject one another, resulting in a dramatic reduction in the particles’ desire to aggregate. The high stability of SeNPs without aggregation may result from the selenium particles’ negative charge. The negative charge is imparted to TA-SeNPs because of the reducing ability of phenolics from the *T. arjuna* bark extract. The negative electrostatic force among TA-SeNPs favors existing in dispersed form. The higher negative charge on the TA-SeNP surface signifies superior stability due to the intrinsic capping by phyto-molecules (Sajadi et al., 2016). There is a correlation between the negative Z-potential values and the negatively charged functional groups from the photochemical in the TA extract on the surface of TA-SeNPs. The presence of carboxyl and hydroxyl groups in the TA bark extract makes them suitable for binding SeNPs (Mellinas et al., 2019). When the total Z-potential of the suspended particles is negative, as seen in this case, they will be repelling each other, favoring staying in a dispersed form. Moreover, the ICP-AES investigation quantified the selenium content to be 82.36 ± 10.2 μg/mL, indicating adequate entrapment in TA-SeNPs.

### Antioxidant activity of TA-SeNPs

**FIGURE 10:**
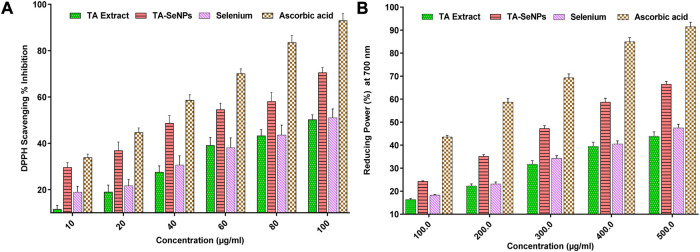
Radical scavenging (DPPH) activity of *Terminalia arjuna* bark extract–reduced SeNPs **(A)** Reducing power activity of *Terminalia arjuna* bark extract–reduced SeNPs **(B)**.

*Terminalia arjuna* bark extract, TA-SeNPs, Se, and ascorbic acid were tested for their ability to scavenge free radicals using the DPPH assay. In a spectrophotometric assay, the ability to scavenge free radicals was quantified by measuring the intensity with which the DPPH color changed from purple to yellow. The concentrations of all solutions (10–100 g/mL) increased the proportion of DPPH scavenging activity. A comparable free radical scavenging activity was observed at 100 μg/mL concentration (Figure 10A). The scavenging action was observed in the following order: ascorbic acid (93.0 ± 3.1) > TA-SeNPs (78.1 ± 2.9) > selenium (51.0 ± 1.8) > TA extracts (50.8 ± 1.6). The antioxidant potential of the TA-SeNPs was significantly higher than that of the TA bark extract, SE, and moderate to standard ascorbic acid. Moreover, the results demonstrated concentration-dependent antioxidant activity, with IC_50_ values for the TA bark extract, TA-SeNPs, selenium, and ascorbic acid of 84.81 ± 0.23 μg/mL, 45.18 ± 0.11 μg/mL, 96.11 ± 0.25 μg/mL, and 12.51 ± 0.16 μg/mL, respectively. The radical scavenging effects and antioxidant activity of extracts were found to be directly proportional to the extract’s phenolic content. A higher concentration of the plant extract showed low free radical scavenging. As a positive control, ascorbic acid was observed to have the best scavenging properties when used at low concentrations. The moderate DPPH radical scavenging activity of the TA-SeNPs is associated with the low IC_50_ value. The results demonstrated that TA-SeNPs possessed higher DPPH radical scavenging activity (*p* < 0.05) than the plant extracts. The reducing power of compounds is measured by their ability to convert Fe^3+^ to Fe^2+^ in the presence of an antioxidant; this causes a shift in the solution’s color from green to blue (Al-Saggaf et al., 2020). In contrast, Perl’s Prussian blue color was formed by powerful reducing agents and absorbed at 700 nm, showing the reducing activities of TA-SeNPs. The amount of absorption of the mixture can predict how strong its reducing power is. The TA-SeNPs could donate electrons to a certain extent. The varying concentrations of the TA bark extract, TA-SeNPs, Se, and ascorbic acid from 200 to 500 μg/mL were assessed for their reducing power activity. At a higher concentration of 500 μg/mL, ascorbic acid (91.51%) and TA-SeNPs (66.51%) demonstrated a maximum reducing power scavenging property when compared to *T arjuna* bark extract (43.92%) and Se (47.51%). The TA-SeNPs showed a much more significant reduction in Fe^3+^ than the TA bark extract and selenium, and was comparable with standard ascorbic acid, as depicted in Figure 10B. It is evident from the results that TA-SeNPs act as effective radical scavengers. Numerous articles suggest that SeNPs have novel biological potential as antioxidants because of their redox modulatory properties (Xia et al., 2022).

### Antibacterial activity of TA-SeNPs

**FIGURE 11:**
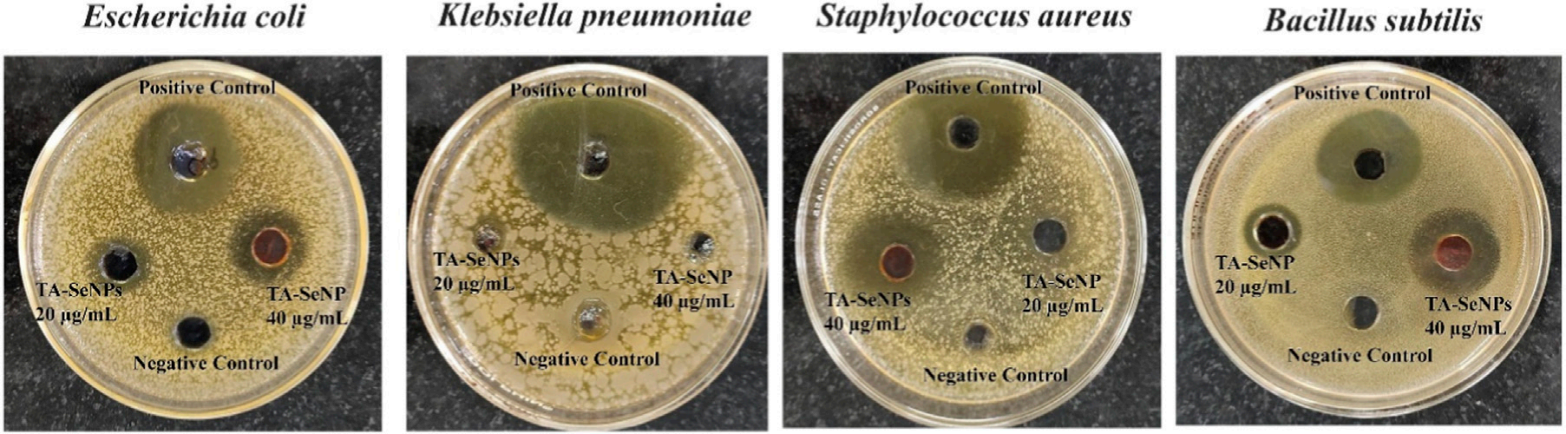
Antibacterial activity of *Terminalia arjuna* bark extract–reduced SeNPs.

Potential pathogenic bacterial strains were used to test the antibacterial efficacy of TA-SeNPs, such as *E. coli*, *K. pneumoniae*, *S. aureus*, and *B. subtilis*; the results are presented in Table 1 and Figure 11. The result revealed that TA-SeNPs can be a potential candidate for suppressing bacterial growth, with variable potency dispensing upon the strain being used. Ciprofloxacin, employed as a standard antibacterial agent at 40 μg/mL, exhibited the largest inhibitory zone against all the strains. It is a potent antibacterial agent in current treatment. The highest zone of inhibition, by TA-SeNPs at 40 μg/mL, was observed against *E. coli* (24.42 ± 0.4 mm) and *B. subtilis* (22.17 ± 0.8 mm). The lowest zones of inhibition were correspondingly noticed against *S. aureus* (18.17 ± 0.2 mm) and *K. pneumoniae* (12.18 ± 0.6 mm). TA-SeNPs displayed a more significant zone of inhibition against *E. coli* and *B. subtilis*, implying that it could be employed as an antibacterial agent. Therefore, biogenic TA-SeNP-fortified formulations can be used against potential pathogens. One mechanism for the antimicrobial potential of SeNPs synthesized from plant extracts is the encapsulation of polyphenolics and other phytochemicals on the SeNPs. These phytochemicals can block the enzymes needed for DNA replication and other forms of gene expansion crucial to a microbe’s survival. In addition, the permeability of the cell wall and/or membrane might be changed by these compounds, which results in the death of pathogens. Possible mechanisms by which selenium NPs exert their antibacterial activity include generating reactive oxygen species and inhibiting enzymes essential to the survival of microorganisms (Indhira et al., 2023).

**TABLE 1:** Antibacterial activity of TA-SeNPs.

Samples	Concentration (µg/mL)	Microorganism			
		*Escherichia coli*	*Bacillus subtilis*	*Klebsiella pneumoniae*	*Staphylococcus aureus*
		Zone of inhibition (mm)			
*Terminalia arjuna* extract	40	10.18 ± 0.3	11.18 ± 0.3	14.36 ± 2.3	00.00 ± 0.0
TA-SeNP	40	24.42 ± 0.4	22.17 ± 0.8	12.18 ± 0.6	18.17 ± 0.2
Selenium	40	11.13 ± 0.5	09.13 ± 0.5	06.13 ± 0.5	12.13 ± 0.5
Ciprofloxacin	40	48.25 ± 0.3	53.53 ± 0.8	36.25 ± 0.2	25.80 ± 0.9

^a^ The data represent the mean of triplicates (±) with a standard deviation (mean ± SD; n = 3).

^b^ Ciprofloxacin (1 mg/mL) *dimethyl sulfoxide (DMSO) (25 μL/well).

### *In vitro* biocompatibility assay

**FIGURE 12:**
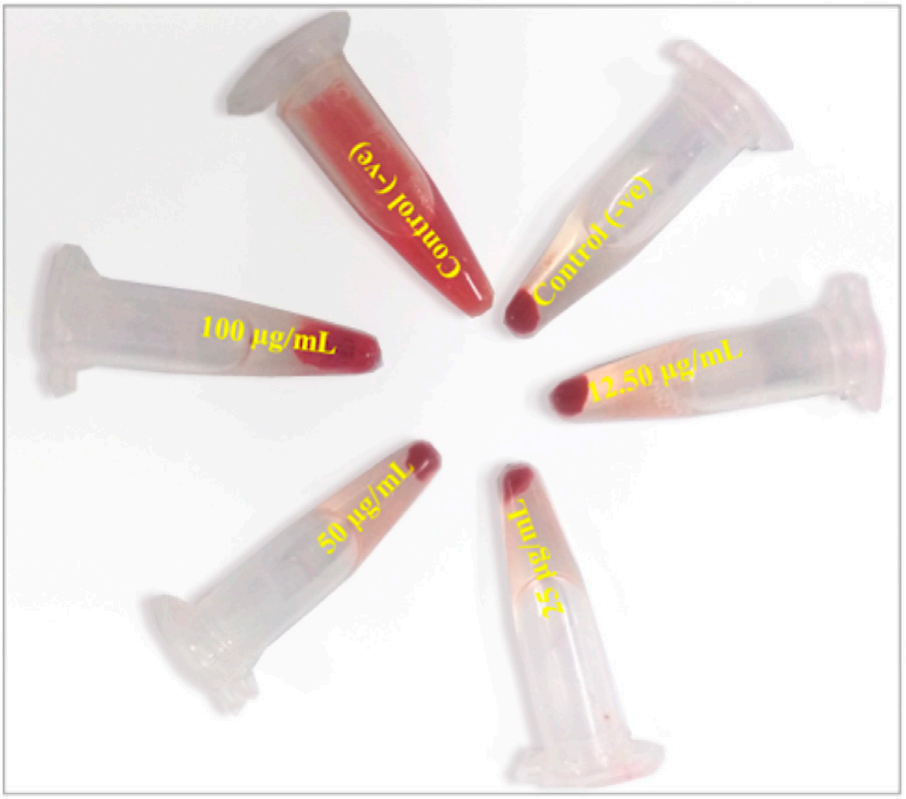
Biocompatibility of *Terminalia arjuna* bark extract–reduced SeNPs against red blood cells.

The cytocompatibility of TA-SeNPs was employed to ensure safety and therapeutic efficacy on application. An active compound must be safe against normal cells, while toxicity against cancer cells is a perquisite for a chemotherapeutic agent. The biocompatibility results on RBCs revealed dose-dependent lysis of red blood cells (Figure 12). A less than 1% value was observed at 12.5 and 25.0 μg/mL; however, a higher lysis of 2.94 ± 0.24 (%) and 4.90 ± 0.98 (%) was observed at 50 and 100 μg/mL, respectively. Hemolysis of <2% is classified as non-hemolytic, between 2% and 5% is classified as slightly hemolytic, and >5% of red blood cell lysis is considered toxic and extremely hemolytic. Moreover, the effect of TA-SeNPs on the viability of HaCaT cells was tested at a concentration range of 100–12.5 μg/mL. The results showed a dose-dependent viability; however, the HaCaT cells were >75% viable at 12.5 and 25 μg/mL, indicating precise use in topical formulations that could inhibit the growth of pathogenic bacteria.

### *In vitro* anticancer activity of TA-SeNPs

**FIGURE 13:**
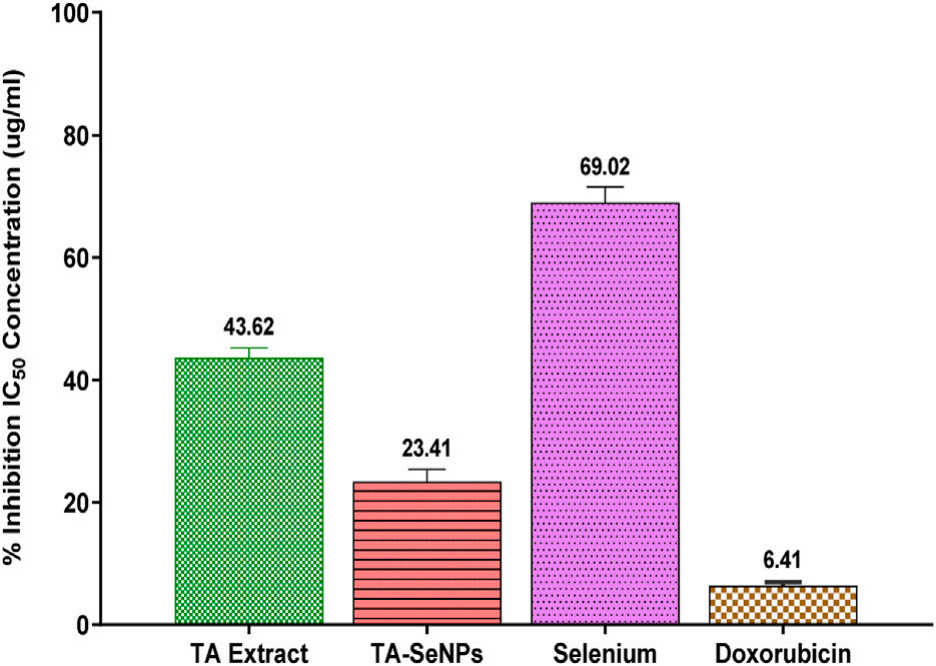
Comparative IC_50_ values *Terminalia arjuna* bark extract–reduced SeNPs against MCF-7 cell lines.

**FIGURE 14:**
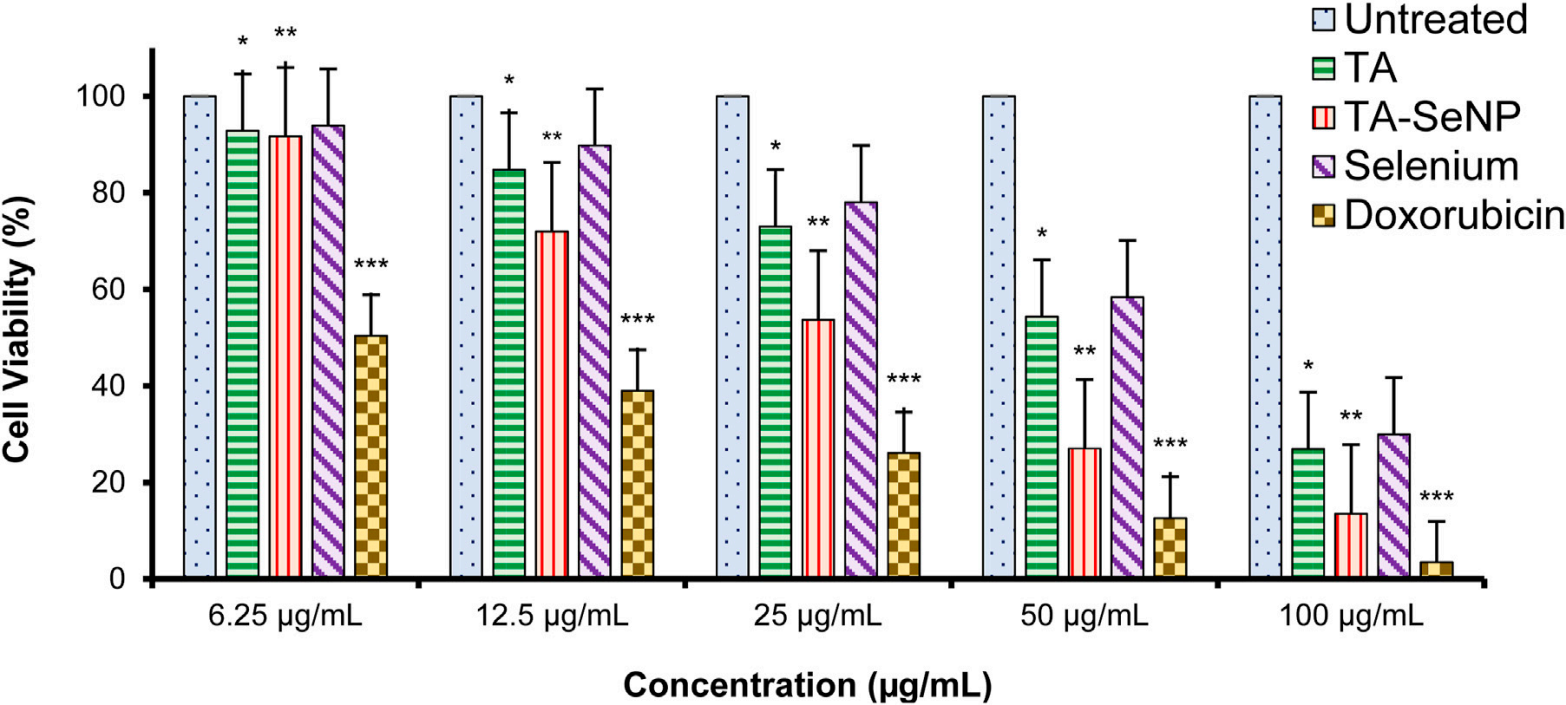
Cell viability (%) of MCF-7 cells treated with the *Terminalia arjuna* bark extract, *Terminalia arjuna* bark extract–reduced SeNPs, selenium, and doxorubicin (n = 3, significant *p-value <0.05, **p-value <0.01, ***p-value <0.001 compared with untreated (control) values represented as mean ± SD).

**FIGURE 15:**
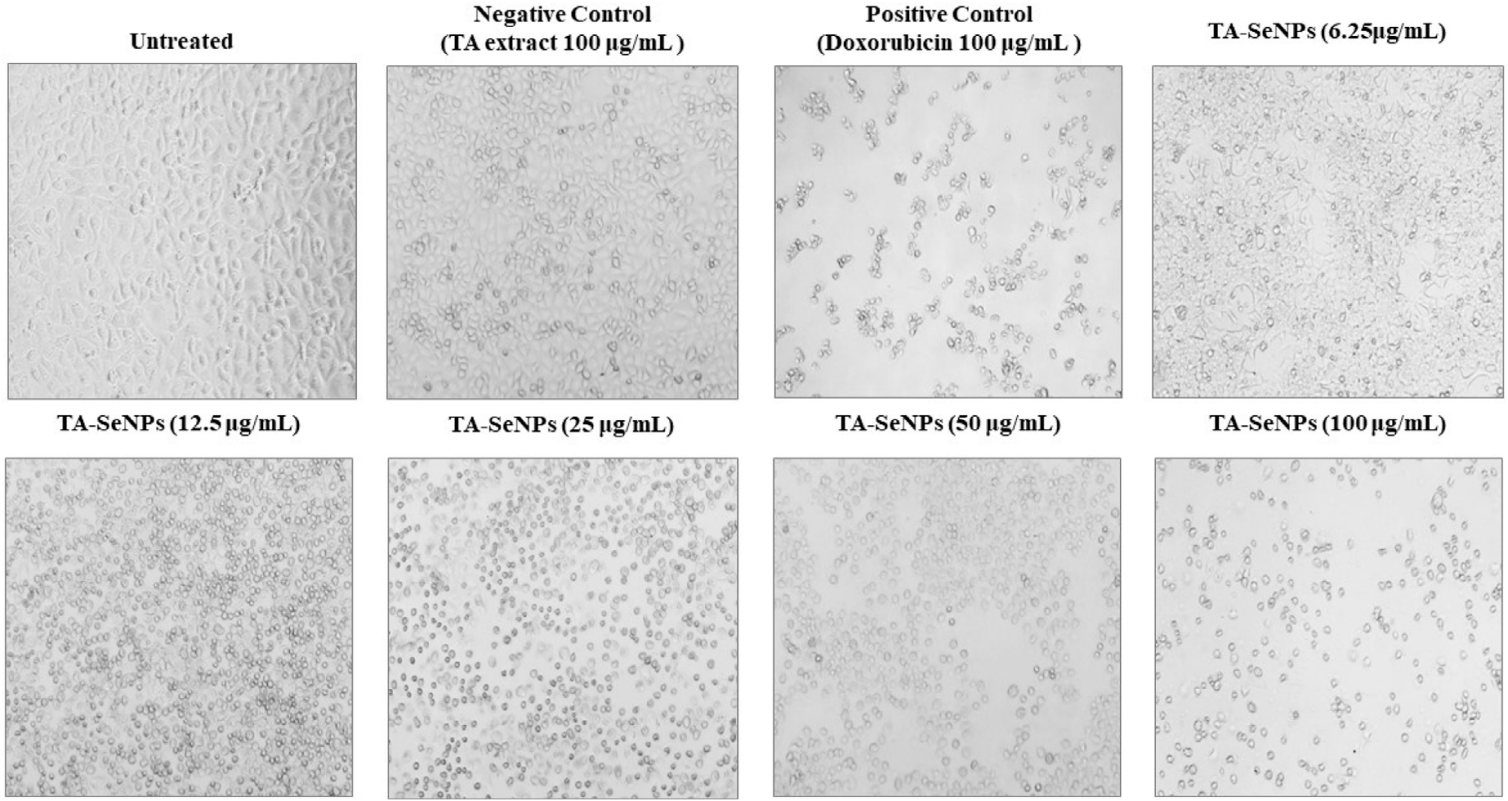
Microscopy image of MCF-7 cells treated with varying concentrations of *Terminalia arjuna* bark extract–reduced SeNPs after incubation of 24 h. Changes in size and shape, divisions between cells close together, and cellular detachments forming round structures are all signs of cytotoxic effects.

The MTT assay against MCF-7 was implemented to investigate the *in vitro* anticancer properties of the TA bark extract, TA-SeNPs, Se, and doxorubicin as the control drug. The test samples, namely, TA-SeNPs and doxorubicin, have significant cytotoxic capability compared to that of the Se and TA bark extract, as presented in Figures 13, 14. The IC_50_ values for the TA bark extract (43.62 μg/mL), TA-SeNPs (23.41 μg/mL), Se (69.02 μg/mL), and standard doxorubicin (6.41 μg/mL) suggest that the TA bark extract and Se displayed moderate cytotoxicity. In contrast, TA-SeNPs demonstrate high cytotoxicity against MCF-7 cells. Treatment of MCF-7 cells with TA-SeNPs significantly suppressed cell growth, indicating good antiproliferative action. Moreover, MTT assays showed that fabricated TA-SeNPs tested at a 12.50 μg/mL concentration produce negligible cytotoxicity (*p* > 0.05 for each). However, the TA-SeNPs at 25, 50, and 100 μg/mL exhibited concentration-dependent cytotoxicity (Figure 14). The cells treated with TA-SeNPs demonstrated decreased viability, loss of cell-to-cell contact, shrinkage, and the formation of apoptotic bodies when observed under the microscope following MTT staining (Figure 15). Microscopical cell morphology analysis revealed that TA-SeNPs were evenly distributed across cancer cell nuclei. Furthermore, the anti-migration investigation demonstrated 18.09 ± 0.98 (%) and 82.01 ± 0.45 (%) for TA-SeNPs and cell alone, respectively, after 36 h (Supplementary Figure S1). Cancer cells treated with TA-SeNPs can exhibit chromosomal instability and mitotic apprehension (MCF-7). Because of their large surface area, TA-SeNPs can be applied as drug delivery vehicles, and NPs can exhibit anticancer potential (Rao et al., 2016). The results show a relationship between the cell viability (%) and cell inhibition (%) for MCF-7 cell lines with the concentration of the fabricated TA-SeNPs used. Selenium has been shown to promote apoptosis in cancer cells while having negligible effects on healthy cells (Khurana et al., 2019). The anticancer activity of SeNPs stems from their ability to increase inflammation and oxidative stress by producing reactive oxygen species (ROS), reducing cell viability. Endocytosis is the primary way SeNPs enter the cells. Malignant cells have redox imbalance where SeNPs generate free radicals, leading to cellular damage. Mitochondrial membrane breakdown and endoplasmic reticulum stress are two examples of these pathways. Protein leakage because of mitochondrial membrane disruption promotes apoptosis through caspase activation. SeNPs disrupt these pathways and inhibit cellular proliferation and tumor microenvironment growth signaling. The culmination of these destructive mechanisms is an end to DNA fragmentation, which causes cell cycle arrest and cell death (Fouda et al., 2022).

### *In vitro* anticancer activity

The antimigration effect of TA-SeNPs against MCF-7 cells was tested at IC_50_ using an *in vitro* scratch assay (Supplementary Figure S1). The results demonstrated that TA-SeNPs significantly inhibited MCF-7 cell migration. The untreated control cell alone showed 67.92 ± 1.70 (%) and 3.27 ± 3.34 (%) of migration after 36 h of treatment, indicating excellent antimigration efficacy. Consistent with some prior investigations, SeNPs were found to inhibit cell migration. Moreover, the study results are in line with those of previous studies that have confirmed the antimigration effect on SeNPs in several different types of cancer cell lines (Othman et al., 2023). Additionally, previous investigations demonstrated that SeNPs dramatically attenuate apoptosis in several cancer cell lines, with MDA-MB-231 cells being the most susceptible and MCF-7 cells being the least sensitive, suggesting that the efficacy of SeNPs may vary depending on the type of cancer cells (Li et al., 2021).

### TA-SeNP-incorporated gel

The TA-SeNP-incorporated gel (Supplementary Figure S2) was tested for pH, viscosity, antimicrobial zone of inhibition, spreadability, extrudability, and swelling index to confirm its suitability in topical biomedical applications. The pH of the TA-SeNP-incorporated gel formulation and control base was 6.8 and 7.2, respectively. The Carbopol 940 gel base alone was transparent and colorless; however, the addition of TA-SeNPs resulted in a dark yellowish-brown color gel. The viscosity of topical semisolid formulation significantly affects the release and diffusion phenomena of fortified active compounds at the site of application. The viscosity study showed a rheology of 6.23 × 10^5^ and 5.98 × 10^5^ cPs for Carbopol 940 alone gel base and TA-SeNP-incorporated gel, respectively. The extrusion and spreadability of the topical formulation from the crimping tube is a customer satisfaction criterion during its application, which depends on polymer concentration, chain length, and polydispersity. The TA-SeNP-incorporated gel and control Carbopol 940 gel base showed a spread time of 10.2 ± 0.12 and 10.19 ± 0.61, respectively, while the percentage extrudability was recorded as 25.76 ± 0.21 and 24.98 ± 0.19 for the TA-SeNP-incorporated gel and control gel base, respectively, indicating that the concentration of Carbopol used in the fabrication of the gel is adequately sufficient. Moreover, the TA-SeNPs incorporated gel and gel base after incubation for 3 h showed a higher volume of swelling (375.99% ± 24.0%) than those with the control gel base (356.98% ± 16.0%), which might be due to its hydrophilic nature, used pH regulator, ionic content, and cross-linking efficacy. A similar study with silver NPs demonstrated 377.97% swelling, indicating that TA-SeNP fortified gel can maintain the hydration property of skin. In addition, the antimicrobial zone of inhibition was 15.29 ± 0.12 (mm), 18.27 ± 0.09 (mm), 20.69 ± 0.16 (mm), and 12.59 ± 0.29 (mm) against *E. coli, K. pneumoniae, S. aureus,* and *B. subtilis*, respectively. These results indicate that the antimicrobial property of TA-SeNP fortified gel is dependent on the release of SeNPs from the polymeric matrix to the surrounding environment. Liu et al. fabricated iprodione-loaded mesoporous SeNPs combined with low melting agarose and investigated its efficacy for strawberry gray mold, which is a fungal disease caused by *Botrytis cinerea*. Plate-based antibacterial tests showed that the colony area of the fabricated SeNPs was far smaller (4.27 m^−2^) than that of the control (25 cm^−2^), with good biocompatibility and improved photosynthetic efficiency of plants that promote plant growth (Liu et al., 2022). Furthermore, the results for contact angle indicating the effect of TA-SeNP addition in the gel base showed a difference in contact angle and surface energy. The gel fortified with TA-SeNPs showed 96.98° ± 0.51, and the gel base alone showed 92.39° ± 0.89°, with a surface energy of 56.22 ± 0.67 and 57.35 ± 0.87, respectively. Furthermore, the stability study indicated smooth physical and texture appearance with maintenance of pH and related properties. In a similar investigation, Jia et al. designed diselenide-cross-linked poly (*N-*vinylcaprolactum) nanogel co-loaded with gold NPs and methotrexate as a multifunctional nanoplatform for improved chemotherapy and computed tomography imaging tumors. The fabricated multifunctional material incorporated within the nanogel showed excellent colloidal stability under physiological conditions, while dissociating rapidly to release the incorporated gold NPs, and methotrexate effectively induces the apoptosis of cancer cells and prevents DNA replication, together contributing to the repolarization of macrophages from the protumor M2-like to antitumor M1-like phenotype *in vitro* (Jia et al., 2023)*.* Therefore, the overall property of TA-SeNPs was retained in gel form and could be a potential candidate for topical biomedical applications. Although selenium has been used for multifarious applications in several experiments, the present investigation reports a green and sustainable process of SeNP fabrication with potential biomedical applications.

## Conclusion

The present investigation outlined a green technique for fabricating SeNPs by a precipitation method utilizing selenious acid and TA bark extract. Successfully synthesizing SeNPs biogenically is achievable due to the presence of biomolecules in the TA bark extract, which was used in the reduction. A variety of multi-spectroscopic techniques confirmed the fabrication of the TA-SeNPs. The TA bark extract exhibited substantial polyphenols confirmed by total phenolic, flavonoid, and tannin contents, which capped and stabilized the TA-SeNPs. Eventually, we conclude that the dose-dependent antioxidant efficacy of the TA-SeNPs containing phytoconstituents opens new avenues for the widespread application of these SeNPs in the pharmaceutical, biomedical, and food industries. TA-SeNPs unveiled a predominant zone of inhibition against representatives of Gram-positive and Gram-negative bacteria. Therefore, we reckon that TA-SeNPs can serve as a potential antibacterial agent for topical applications in incorporated gel form. The work further suggests that surface functionalization with SeNPs might be a safe, effective antioxidant, antibacterial, and cytotoxic strategy and can be a novel therapeutic agent. Therefore, the burgeoning interest in developing new nanomaterials derived from selenium will expand in the coming year.

## Data Availability

The original contributions presented in the study are included in the article/Supplementary Material; further inquiries can be directed to the corresponding authors.
