# Correction: Facile green synthesis and characterization of *Terminalia arjuna* bark phenolic–selenium nanogel: a biocompatible and green nano-biomaterial for multifaceted biological applications

**DOI:** 10.3389/fchem.2025.1743284

**Published:** 2026-01-08

**Authors:** Abhijeet Puri, Popat Mohite, Swati Patil, Vijay R. Chidrawar, Yogesh V. Ushir, Rajesh Dodiya, Sudarshan Singh

**Affiliations:** 1 St. John Institute of Pharmacy and Research, Palghar, Maharashtra, India; 2 Department of Pharmacognosy, Principal K. M. Kundnani College of Pharmacy, Mumbai, Maharashtra, India; 3 SVKM’s NMIMS School of Pharmacy and Technology Management, Jadcharia Telangana, India; 4 SMBT College of Pharmacy and Institute of Diploma Pharmacy, Nashik, Maharashtra, India; 5 School of Pharmacy, Faculty of Pharmacy, Parul University, Waghodia, Gujarat, India; 6 Department of Pharmaceutical Sciences, Faculty of Pharmacy, Chiang Mai University, Chiang Mai, Thailand; 7 Office of Research Administration, Chiang Mai University, Chiang Mai, Thailand

**Keywords:** antioxidant, antibacterial, anticancer, gel, green synthesis, selenium nanoparticle, *Terminalia arjuna*

In the published article, there was an error in Figure 11 as published. While submitting the manuscript and figures, there was an overlap between the antibacterial zone of inhibition images in the present publication and the images in a previous publication reporting similar experiments and results, which resulted in the incorrect figures being included in this article. The corrected Figure 11 and its caption appear below.

**FIGURE 11:**
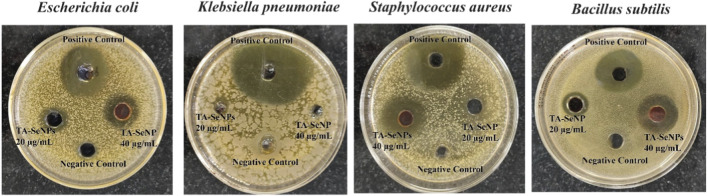
Antibacterial activity of *Terminalia arjuna* bark extract–reduced SeNPs.

The original article has been updated.

