# Supplementary material for: Facile green synthesis and characterization of Terminalia arjuna bark phenolic–selenium nanogel: a biocompatible and green nano-biomaterial for multifaceted biological applications
Source: Front Chem. 2023 Sep 22;11:1273360. doi: 10.3389/fchem.2023.1273360 (PMC10556707; doi:10.3389/fchem.2023.1273360)
Supplement: Supplementary file 1 [file DataSheet1.docx]

**Facile Green Synthesis and Characterization of Terminalia arjuna Bark Phenolic-Selenium Nano Gel: A Biocompatible and Green Nanobiomaterial for Multifaceted Biological Applications**

Abhijeet Puri ^1^*, Popat Mohite ^1^, Swati Patil ^2^, Vijay R Chidrawar ^3^, Yogesh V Ushir ^4^, Rajesh Dodiya^5^, Sudarshan Singh ^6^*

^1^ St. John Institute of Pharmacy and Research, Palghar, 401404, Maharashtra, India; [AP: [abhijeetp@sjipr.edu.in](mailto:abhijeetp@sjipr.edu.in); PM: [mohitepb@gmail.com](mailto:mohitepb@gmail.com)]

^2^ Department of Pharmacognosy, Principal K. M. Kundnani College of Pharmacy, Mumbai, 400005, Maharashtra, India; [SP: [ss.patil@kmkcp.edu.in](mailto:ss.patil@kmkcp.edu.in)]

^3^ Department of Pharmacology, SVKM’s NMIMS School of Pharmacy and Technology Management, Jadcharia 509301 Telangana, India; [VRC: [vijay.chidrawar@gmail.com](mailto:vijay.chidrawar@gmail.com)]

^4^ SMBT College of Pharmacy and Institute of Diploma Pharmacy, Nashik 422403, Maharashtra, India; [YUV: [ushir29@gmail.com](mailto:ushir29@gmail.com)]

^5^ School of Pharmacy, Faculty of Pharmacy, Parul University, Waghodia, Gujarat 391760, India; [RD: [rajesh.dodiya24770@paruluniversity.ac.in](mailto:rajesh.dodiya24770@paruluniversity.ac.in)]

^6^ Department of Pharmaceutical Sciences, Faculty of Pharmacy, Chiang Mai University, Chiang Mai 50200, Thailand; [SS:[sudarshansingh83@hotmail.com](mailto:sudarshansingh83@hotmail.com)]

*Corresponding authors

Sudarshan Singh ([sudarshansingh83@hotmail.com](mailto:sudarshansingh83@hotmail.com))

Abhijeet Puri([abhijeetp@sjipr.edu.in](mailto:abhijeetp@sjipr.edu.in))


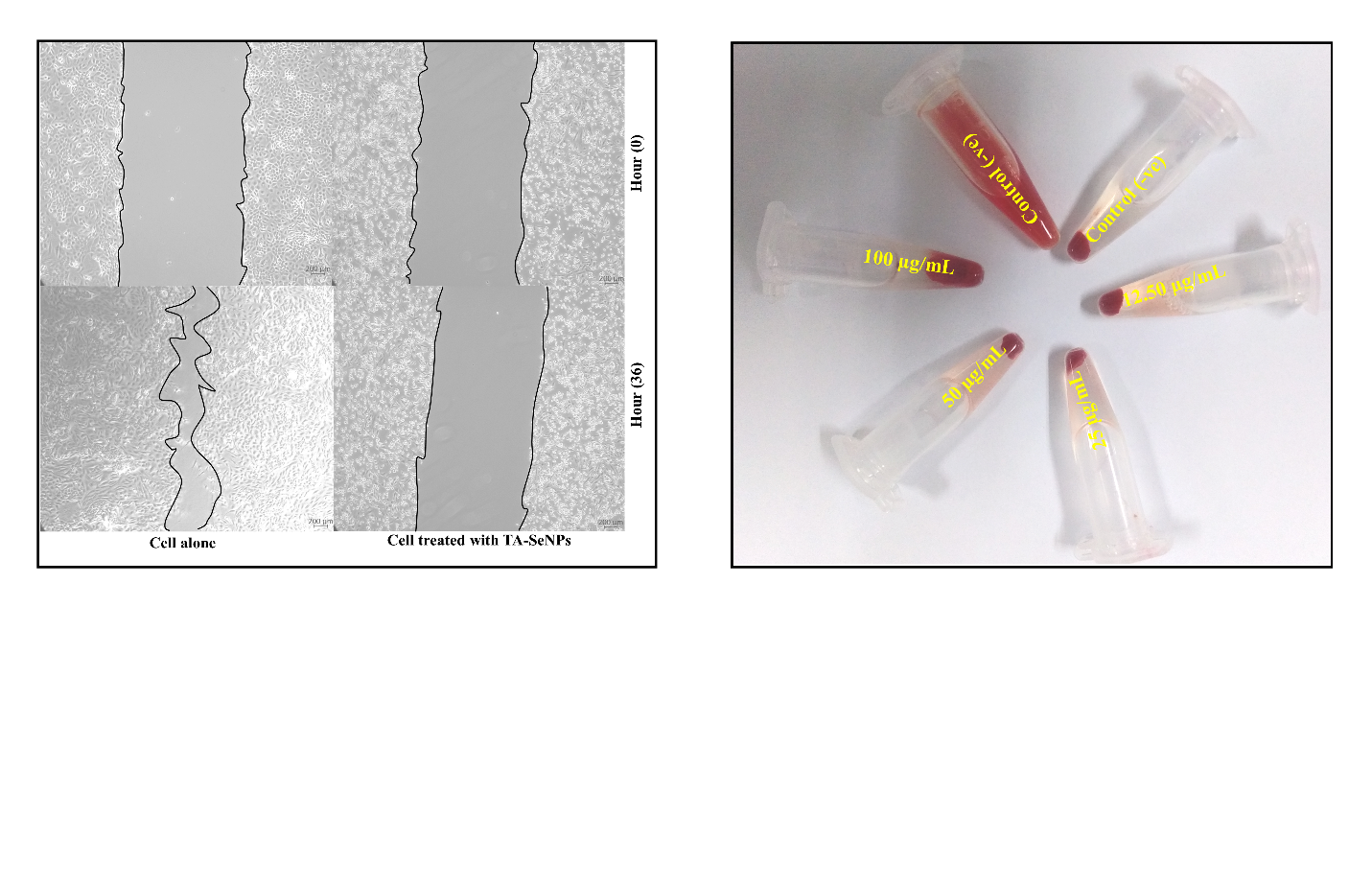


**Figure S1.** Anti-proliferative effect of MCF 7 cells in presence of TA-SeNPs


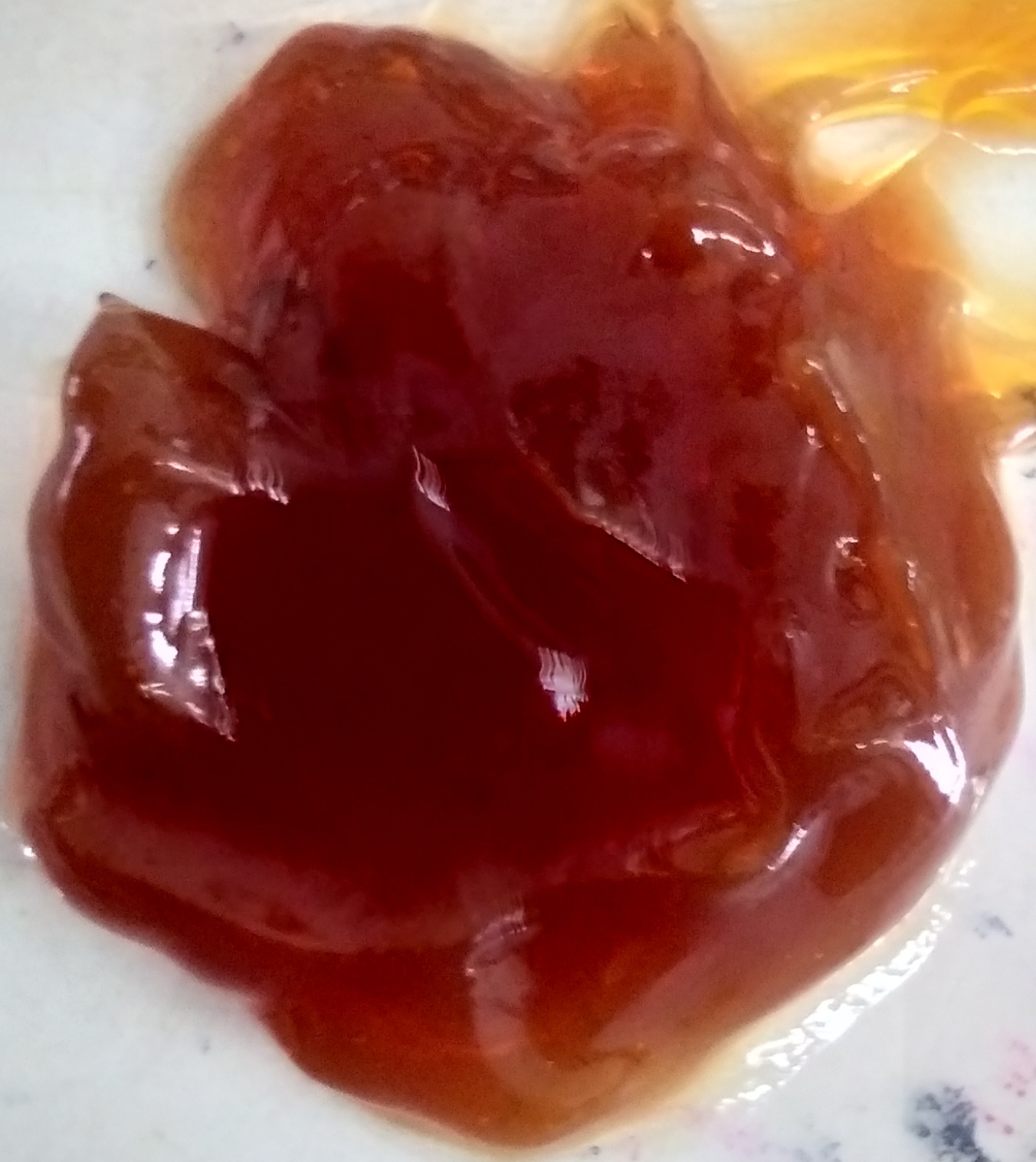


**Figure S2.** Carbopol gel incorporated TA-SeNPs
